# Repurposed Leather with Sensing Capabilities for Multifunctional Electronic Skin

**DOI:** 10.1002/advs.201801283

**Published:** 2018-12-01

**Authors:** Binghua Zou, Yuanyuan Chen, Yihan Liu, Ruijie Xie, Qinjie Du, Tao Zhang, Yu Shen, Bing Zheng, Sheng Li, Jiansheng Wu, Weina Zhang, Wei Huang, Xin Huang, Fengwei Huo

**Affiliations:** ^1^ Key Laboratory of Flexible Electronics (KLOFE) & Institute of Advanced Materials (IAM) Nanjing Tech University (NanjingTech) 30 South Puzhu Road Nanjing 211816 P. R. China; ^2^ Shaanxi Institute of Flexible Electronics (SIFE) Northwestern Polytechnical University (NPU) 127 West Youyi Road Xi'an 710072 P. R. China; ^3^ National Engineering Laboratory for Clean Technology of Leather Manufacture Sichuan University Chengdu 610065 P. R. China

**Keywords:** electronic skin, flexible, leather, sensing capability, wearable

## Abstract

Electronic skin (e‐skin), an important part toward the realization of artificial intelligence, has been developing through comprehending, mimicking, and eventually outperforming skin in some aspects. Most of the e‐skin substrates are flexible polymers, such as polydimethylsiloxane (PDMS). Although PDMS was found to be biocompatible, it is not suitable for long‐time wearing due to its air impermeability. This study reports a simple and designable leather based e‐skin by merging the natural sophisticated structure and wearing comfort of leather with the multifunctional properties of nanomaterials. The leather based e‐skin could make leather, “the dead skin,” repurposed for its sensing capabilities. This e‐skin can be applied in flexible pressure sensors, displays, user‐interactive devices, etc. It provides a new class of materials for the development of multifunctional e‐skin to mimic or even outshine the functions of real skin.

Nature provides efficient strategies to achieve certain functions by “designing” sophisticated structure. Skin, outer covering of the body, serves as a physical barrier to protect inner organs and possesses a neural network to sense environmental stimuli, such as temperature, vibration, pressure, etc.^1^, ^2^, ^3^, ^4^ Electronic skin (e‐skin) is an artificial skin that mimics the functions of human skin. To develop e‐skin that understands, emulates, or even outperforms human skin is motivated by the promise of creating autonomous artificial intelligence, medical diagnostics, and biomimetic prosthetics.^5^, ^6^, ^7^, ^8^, ^9^, ^10^, ^11^ In recent years, significant progress has been witnessed in the development and advancement of e‐skin.^12^, ^13^, ^14^, ^15^, ^16^, ^17^, ^18^, ^19^, ^20^, ^21^, ^22^, ^23^, ^24^, ^25^, ^26^, ^27^, ^28^, ^29^, ^30^, ^31^, ^32^, ^33^, ^34^, ^35^, ^36^, ^37^ Bao and co‐workers have pioneered many strategies to innovate the performance of e‐skin. They demonstrated that by introducing microstructured polydimethylsiloxane (PDMS) films, the obtained e‐skin can be endowed with unprecedented sensitivity and short response time.^29^, ^30^, ^31^, ^32^, ^33^ Additionally, flexibility is a crucial property for e‐skin to mimic the mechanical property of human skin. By combining filamentary serpentine nanoribbons with elastomeric substrate, quite a few inspiring approaches have been developed by Rogers and co‐workers to transform traditionally brittle materials into highly flexible, stretchable, and performable e‐skin.^34^, ^35^, ^36^ Notably, e‐skin that rationally integrates different kinds of functions can realize and even outperform the performance of real skin. Someya and co‐workers have reported ultraflexible organic optical systems. They used ultrathin film as the substrate to incorporate different types of organic devices, thereby endowing e‐skin with multiple electronic functionalities, such as sensors and displays,^37^ etc. In a word, finding a responsible substrate that is flexible, well‐structured, and comfortable is essential to achieve multifunctional e‐skin.

Leather is a traditional natural material obtained from animal skin, meanwhile it inherits the sophisticated structure from the skin.^38^ The traditional leather recovers the flexibility of skin, while its sensing capability is still under deprivation, an important function of real skin.^39^, ^40^, ^41^ Rogers and co‐workers used PDMS as a glue to bind their silicon devices on leather but ignored the merits of leather structures and properties by treating it only as a simple substrate.^42^ The hierarchical structure of leather makes it easy to load other materials and as a potential candidate to fabricate high performance e‐skin. We believe that, by combining different functional materials with leather, it is possible to make leather, “the dead skin,” repurpose or even outperform the properties of real skin.

Herein, we report a new designed e‐skin, a leather based e‐skin that integrates leather with different kinds of functional materials, such as carbon nanotubes (CNTs), silver nanowires (Ag NWs), to endow the leather with sensing capabilities (**Scheme** **1**). In the leather based e‐skin, leather with sophisticated hierarchical structure serves as a unique platform to load different kinds of functional nanomaterials. Nanomaterials with multifunctional properties^43^ can convert the external stimuli to different signals in a way similar to the role of sensory nerve in real skin. Based on the above design, the prepared leather based e‐skin can be used as flexible pressure sensor, display, and user‐interactive device. Notably, the fabrication process of the leather based e‐skin is simple, general, and scalable, and it also matches well with the tanning and dying procedures in the traditional leather industry. With rational design, leather can be repurposed and even outdo the features of real skin, providing a new platform for the realization of multifunctional e‐skin.

**Scheme 1:**
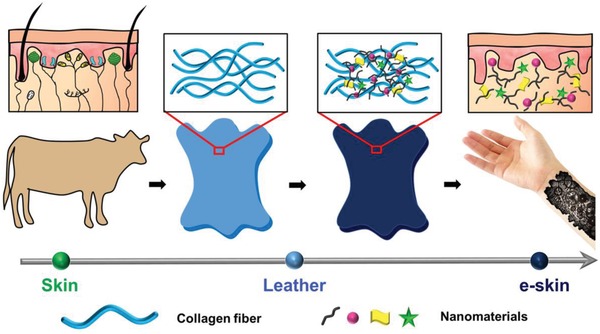
Schematic of design principle of leather based e‐skin. By merging the sophisticated hierarchical structure of leather with the multifunctional properties of nanomaterials, to repurpose leather with sensing capability similar to real skin and even outperform skin. Light blue material denotes the pure leather; dark blue material is leather based e‐skin.

Sensing external stimuli and then converting the stimuli into analog electronic signals is the major function of e‐skin. Therefore, conductivity is necessary for the leather based e‐skin to repurpose the sensing capabilities of real skin. Through functionalized with conductive materials, the leather can be conductive. Here, leather and acid treated carbon nanotubes (a‐CNTs) were taken as an example to fabricate conductive leather. Flexible as it is, leather possesses a sophisticated hierarchical structure from nanoscale to macroscale. This should be attributed to its hierarchical structure: amino acids, collagen molecule, collagen fibril (nm), collagen fiber (µm), and leather (cm).^40^ Such structure makes leather a promising candidate for loading and/or being modified by other materials. CNTs have been widely used for e‐skin due to their exceptional electronic and mechanical properties.^44^, ^45^, ^46^ a‐CNTs used here are synthesized by following the modified Hummers method due to their good dispersion in water (Figure S1, Supporting Information).^47^ The a‐CNTs, tens of micrometers in length, are mechanically robust and flexible (Figure S2, Supporting Information). In addition, they have the average diameter of 30 nm, much smaller than the space among collagen fibers of leather (µm–mm), thereby ensuring their easy permeation into the hierarchical network of the leather. Based on the above, the conductive leather can be fabricated by filtrating a‐CNTs using leather as a filter, and then the a‐CNTs distributed well inside the hierarchical structure of leather (**Figure** **1**a). Both photographs and scanning electron microscope (SEM) images demonstrated the well permeation of a‐CNTs into the structure of leather (Figure 1b–e). By increasing the usage amount of a‐CNTs (0.08, 0.16, and 0.32 mg cm^−2^), the colors of the samples changed from grey to dark black (inset images in Figure 1f). The SEM images could also certify the increasing amount of a‐CNTs in the leather (Figure S3, Supporting Information). Accordingly, the conductivities of leathers could be tuned to 173.73 ± 11.96, 25.13 ± 2.89, and 4.39 ± 0.68 kΩ sq^−1^, respectively (Figure 1f). Besides, the conductive leather can be bent into different angles while still maintaining good conductivity (Figure S4, Supporting Information). This should be ascribed to the intrinsic flexible property both of leather and a‐CNTs.

**Figure 1:**
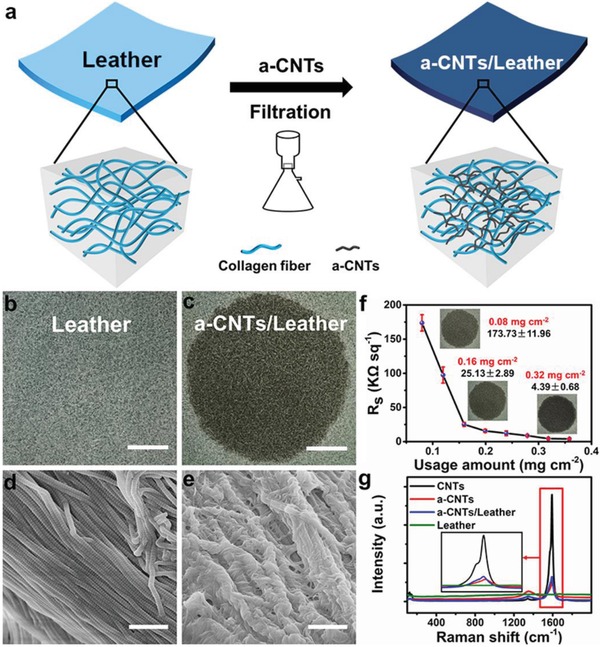
Conductive leather. a) The fabrication process of conductive leather. Leather is taken as filter and filtrating with a‐CNTs, to fabricate conductive leather. Photographs of b) leather, c) conductive leather, and SEM images of d) leather, e) conductive leather. f) Tunable conductivity of conductive leather and photographs of conductive leather after filtrating with 0.08, 0.16, and 0.32 mg cm^−2^ a‐CNTs, respectively. g) Raman spectrum of CNTs, a‐CNTs, leather, and conductive leather. Scale bars in (b),(c) 1 cm; Scale bars in (d),(e) 1 µm.

It is believed that two main reasons are accountable for the well permeation of a‐CNTs in leather. First, the hierarchical and porous structure of leather (Figure S5, Supporting Information). It is well known that leather contains multiscale voids, ranging from tens of nanoscale to sub millimeter scale.^40^ Therefore, a‐CNTs with small size (30 nm in diameter) and flexible property can permeate into leather successfully. Second, the chemical interaction between leather and a‐CNTs. There are abundant amino residues on each peptide chain of collagen fibril, proving leather with full of active groups, such as —COOH, —NH_2_, —OH. Meanwhile, a‐CNTs own lots of —COOH and —OH active groups. Hence, hydrogen bonding might be formed between a‐CNTs and leather. Further evidence can be found in Raman studies on the CNTs, a‐CNTs, and a‐CNTs/leather samples. In Raman spectrum, the G band of a‐CNTs/leather showed a red shift compared with a‐CNTs (Figure 1g), suggesting the existence of chemical interaction between a‐CNTs and leather.^48^ In short, the well permeation of a‐CNTs into leather was a result from both the structure of leather and the chemical interaction between leather and a‐CNTs.

Based on the two reasons above, such fabrication approach can also be extended to other nanomaterials (**Figure** **2**), such as Ag NWs, graphene oxides (GO), poly(3,4‐ethylenedioxythiophene) nanofibers (PEDOT NFs), as well as gold nanoparticles (Au NPs). It demonstrated that this approach can combine different functional materials with leather. By tuning the usage amount of functional materials, leather can be endowed with different functions. For instance, by tuning the usage amount of Ag NWs from 0.08 to 0.16 to 0.32 mg cm^−2^, the obtained leather exhibited conductivities from 113.13 ± 22.13 to 15.52 ± 2.08 to 6.15 ± 0.71 Ω sq^−1^, respectively (Figures S6 and S7, Supporting Information). It indicated that by combining the leather with different kinds of functional nanomaterials and different usage amounts, e‐skin with various functions could be gained on purpose. In addition, the fabrication process was scalable and matched with the tanning and dying procedures in the leather industry thereby providing a low‐cost approach for mass production (Figure S8, Supporting Information). The resulted material can act as a multifunctional e‐skin, such as flexible pressure sensor, display, and user‐interactive device.

**Figure 2:**
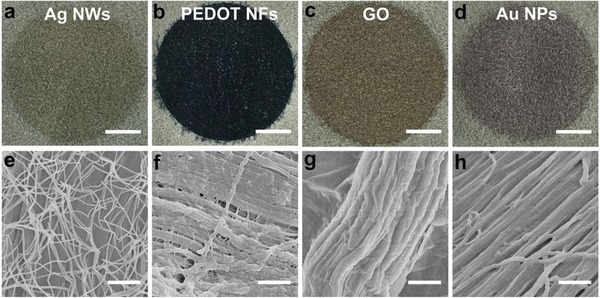
The fabrication strategy can be extended to other nanomaterials. Photographs and SEM images of leather filtrated with a,e) Ag NWs, b,f) PEDOT NFs, c,g) GO, d,h) Au NPs, respectively. Scale bars in (b)–(d) 1 cm; Scale bars in (e)–(h) 1 µm.

Based on the conductive leather (a‐CNTs/leather), we designed a flexible and wearable pressure sensor with repurposed sensing property. The pressure sensor was fabricated using one conductive leather and one leather patterned with interdigitated electrode arrays. The two leathers were stitched together by sewing machine due to their tailorable property (**Figure** **3**a). The key component of this pressure sensor was a conductive leather, the average conductivity of which was 15.70 ± 2.53 kΩ sq^−1^.

**Figure 3:**
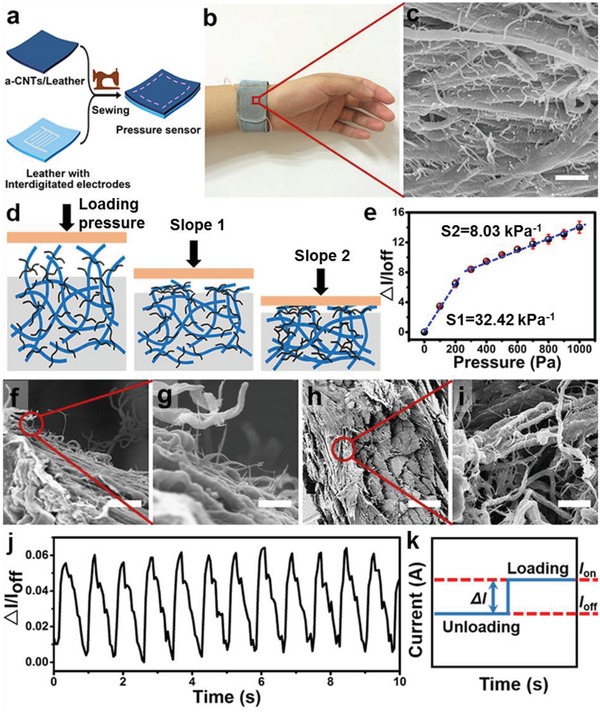
Flexible and wearable pressure sensor based on conductive leather. a) Schematic illustration of the fabrication procedure of flexible pressure sensor. By stitching one conductive leather and one leather patterned with interdigitated electrode arrays together with a sewing machine, to fabricate pressure sensor based on conductive leather. b) Photograph of the watchband shaped e‐skin directly above the artery of the wrist. c) SEM image of the cross section morphology of a‐CNTs/leather. d) A schematic diagram of a‐CNTs/leather under different pressures. e) Current responses to various pressures. f,g) SEM images of the surface morphology of a‐CNTs/leather at different magnifications. h,i) SEM images of the cross‐section of a‐CNTs/leather at different magnifications. j) Measurement of the physical force of wrist pulses under normal condition (≈72 beats min^−1^). k) Sensing mechanism: current changes in response to loading and unloading pressure (*I*_off_: unloading, *I*_on_: loading). Scale bars in (c),(g) 500 nm; Scale bars in (f) 2 µm; Scale bars in (h) 200 µm; Scale bars in (i) 1 µm.

The sensing mechanism of the pressure sensor is that the conductive pathways among the interdigitated electrode arrays were dependent on the external loaded force. Under certain pressure, the a‐CNTs will be piled up, enabling more a‐CNTs to contact with each other and resulting in an increase in current when applying a fixed 3 V (Figure 3k). In addition, the device can be tailored into different shapes due to the intrinsic wearable and tailorable properties of leather.

For human skin, sense of touch involves arrays of different mechanoreceptors in order to achieve a high sensitivity and a wide range of pressure. It has been found that Tactile Corpuscles and Pacinian Corpuscles are responsible for sensing light touch and gross pressure change, respectively.^3^, ^4^ Leather naturally inherits the hierarchical structure from real skin, which has a rough surface (Figure 3f,g) and sophisticated inner parts (Figure 3h,i), providing the possibility to detect different kinds of pressure sensing with just one design. The sensitivity of this pressure sensor can be defined as: *S* = (Δ*I*/*I*_off_)/Δ*P*, where Δ*I* is the relative change in current, *I*_off_ the current of the sensor with only base pressure and Δ*P* the change in applied pressure.

The flexible pressure sensor exhibited a sensitivity high of 32.42 kPa^−1^ when the pressure was lower than 200 Pa (slope 1 in Figure 3e). Such sensitivity is probably a result of the synergistic effect between the a‐CNTs on the surface of a‐CNTs/leather (Figure 3d) that touched the finger electrodes and the slightly compressive deformation of a‐CNTs/leather. When the pressure ranging from 200 to 1000 Pa (slope 2 in Figure 3e), the sensitivity was 8.03 kPa^−1^ and generated mainly by the compressive deformation of a‐CNTs/leather (Figure 3d). The photographs of a‐CNTs/leather under different pressures (Figure S9, Supporting Information) further demonstrated that the surface structure of a‐CNTs/leather contributes to the slope 1. The sensitivity of this pressure sensor has achieved a high sensitivity as previously reported e‐skin made by other materials (Table S1, Supporting Information). In addition, the pressure sensor could immediately respond to a light feather of 32.5 mg and showed a fast response time of 40 ms (Figure S10, Supporting Information). Similar to the behavior of pressure sensing capability of real skin, the leather based e‐skin showed high sensitivity mode of “light touch” and wide range mode of “deep press,” respectively. The leather based e‐skin could also reveal different response times and peak shapes under various kinds of forces, such as bend or torsion (Figure S11, Supporting Information), proving its potential in detecting other forces. We further demonstrated the durability of this pressure sensor under a pressure of 2.5 kPa (Figure S12, Supporting Information). The signal‐to‐noise ratios were well maintained and the current amplitude exhibited negligible changes after 8000 loading/unloading cycles.

This flexible pressure sensor is wearable and can be shaped into a watchband (Figure 3b,c) to monitor the wrist pulse of human in real time. Wrist pulse is an important indicator of arterial blood pressure and heart rate, and also provides a great number of valuable information for medical diagnosis.^49^ For instance, some cardiovascular diseases are asymptomatic in the initial phase but can result in pathological pulse and impact arterial blood pressure. Therefore, continuously monitoring the blood pressure of human artery by wrist pulse can provide a rapid and noninvasive way for diseases diagnosis. The pressure sensor watchband can read out the wrist pulses precisely of a healthy person (Figure 3j about 72 beats min^−1^). It can also clearly collect the typical characteristics of wrist pulses, including percussion wave (P‐wave), tidal wave (T‐wave), valley, and diastolic wave (D‐wave) (Figure S13, Supporting Information). That means this pressure sensor can identify the impalpable differences in wrist pulses. Moreover, leather based devices are suitable for long term wearing, and will not cause discomfort as polymer substrates do (Figure S14, Supporting Information).

By merging the unique hierarchical structure of leather with excellent properties of nanomaterials, the a‐CNTs/leather based e‐skin can not only measure different pressures but also be used to monitor human's wrist pulse continuously. It demonstrates that by rational design, leather based e‐skin can repurpose the sense of touch.

Display is important to improving the communication and lifestyle of people, and it significantly promotes the visualization of information. Benefiting from its plane structure and the tunable conductivity, a‐CNTs/leather can be used as the back electrode for display. The structure of the display device was illustrated in **Figure** **4**a. The display device was fabricated on the surface of conductive leather with an average conductivity of 4.39 ± 0.68 kΩ sq^−1^ by coating electroluminescence layer. As proof‐of‐concept, commercial available inorganic material (ZnS:Cu, Figure S15, Supporting Information) was chosen as an electroluminescence layer. Ag NW was used as a transparent electrode for the display because of its electronic, optical, and flexible mechanical properties. Because Ag NW electrode was drawn by pen, the device can display diverse patterns. Here, a flower was drawn on the device by Ag NWs and it exhibited a bright light as the power on (Figure 4b,c). In addition, conductive leather based on other nanomaterials can also be applied in display, for example, Ag NWs/leather (Figure S16, Supporting Information).

**Figure 4:**
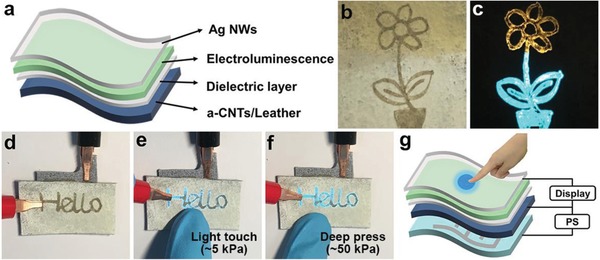
Leather based display and user‐interactive e‐skin provide an instantaneous visual response. a) Structure of leather based display. b,c) Photographs of leather based display before and after being turned on, respectively. d–f) Photographs showed that by tuning the applied force from light touch e) to deep press, f) the brightness of display increased simultaneously. g) Structure of leather based user‐interactive e‐skin.

Display is adept in visualizing information, so integrating the display with the presynthesized flexible pressure sensor can form an e‐skin, which possesses both interactive brightness changing and pressure sensing properties. A flexible pressure sensor was mounted on finger pulps and a display on the back of the hand, which were connected to each other. When given a weak handshake (≈5 kPa), the star was light blue. Whereas given a strong handshake (≈100 kPa), the star turned to bright blue (Figure S17, Supporting Information). The change in the brightness can be visually seen from the inset photographs of the display in Figure S18 in the Supporting Information. With the increase in pressure, the growth tendency of the current is similar to that of the brightness. This demonstrated the feasibility of visualizing information of pressure in the form of brightness changing.

Due to the hierarchical and porous structure through the whole leather, a user‐interactive e‐skin can be achieved by combining single piece of a‐CNTs/leather with a piece of interdigitated electrode arrays patterned leather. The a‐CNTs/leather not only played as an active material of the pressure sensor, but also the back electrode of the display. The user‐interactive device was able to provide an instantaneous visual response by directly mapping pressure to light. The schematic configuration and photograph of the integrated system was shown in Figure 4g,d, respectively. The brightness change of the display was dependent on the applied pressure. By tuning the applied force from a light touch (5 kPa) to a deep press (50 kPa), the word “Hello” changed from light blue to bright blue (Figure 4e,f). This interactive e‐skin was capable of spatially mapping and responding to the applied pressure simultaneously (Figure S19, Supporting Information). Such multifunctional e‐skin plays a decisive role in the development and advancement of artificial intelligence.

In summary, we have demonstrated a practical e‐skin material, which is capable of repurposing the pressure sensing property of skin and even outperformed it in display, user‐interaction, as well as human health monitoring. This e‐skin was accomplished based on the features of leather and nanomaterials, i.e., the sophisticated structure of leather and the multifunctional properties of nanomaterials. Leather as a byproduct of the meat industry has long been used as a comfortable wearable material since ancient times. Benefiting from the sophisticated hierarchical structure of leather, the leather based e‐skin can achieve a high sensitivity. The fabrication process of leather based e‐skin was simple, general, and scalable, similar to those widely used for tanning and dying in the leather industry, thereby providing a low‐cost approach for mass production. This strategy using leather as a substrate for flexible and wearable electronics demonstrates a value‐added perspective of up‐cycling of low value leather. We believe that such design will lead to the emergence of new flexible electronics and contribute to unleashing the potentials of multifunctional e‐skin, such as intelligent robot and health monitoring devices.

## Experimental Section

*Preparation of a‐CNTs*: A‐CNTs were synthesized by the oxidation of CNTs with the modified Hummer's method. First, CNT powder (2 g) and sodium nitrate (1 g) were added into the concentrated sulphuric acid (46 mL) and stirred in an ice bath. Then, potassium permanganate (6 g) was slowly added into the suspension of the ice bath. The suspension was stirred for more than 8 h at 30 °C. Distilled water (92 mL) was slowly added and then heated up to 95 °C. After 15 min, the suspension was further diluted with warm distilled water (280 mL). Hydrogen peroxide (10 mL) was added and the mixture was washed with hydrochloric acid (1000 mL 5% wt) and the a‐CNTs were separated by centrifugation.

*Preparation of Conductive Leather*: Cow split leather was provided by the National Engineering Laboratory for Clean Technology of Leather Manufacture, Sichuan University. The leather was filtrated by the prepared a‐CNTs (0.2 mg mL^−1^) with different usage amounts. Ten samples for each usage amount. Finally, the samples were dried on the oven for 6 h at 60 °C. This approach is applicable to other types of leathers.

*Fabrication of Flexible and Wearable Pressure Sensor*: The interdigitated electrodes were drawn by a pen and Ag NWs aqueous solution (10 mg mL^−1^) was the ink. The space between the adjacent electrodes was 2 mm, and the interdigitated electrodes were 2 mm in width. Then the leather patterned interdigitated electrode arrays and the prepared conductive leather were stitched together by a sewing machine to fabricate flexible and wearable pressure sensor. By tailoring the conductive leather into a watchband shape, a watchband pressure sensor was fabricated.

*Fabrication of Leather Based Display*: A‐CNTs/leather was taken as the back electrode of the display. The dielectric layer was coated on the surface of a‐CNTs/leather. Then the electroluminescence layer (ZnS:Cu) was coated on the dielectric layer. Finally, the Ag NW (10 mg mL^−1^) was taken as the transparent electrode and a pen was used to draw patterns on the electroluminescence layer.

*Fabrication of User‐Interactive Device*: The user‐interactive device was fabricated by taking a single piece of a‐CNTs/leather as the active material of the pressure sensor and the back electrode of the display. The dielectric layer was coated on the surface of a‐CNTs/leather. Then the electroluminescence layer (ZnS:Cu) was coated on the dielectric layer. Ag NWs (10 mg mL^−1^) were taken as the transparent electrode and a pen was used to draw the letter “Hello” on the electroluminescence layer. Finally, the above material was glued with leather that was patterned with interdigitated electrode arrays.

*Device Characterization*: SEM was performed using a Hitachi S4800 with a 5 kV accelerating voltage. Transmission electron microscope images were taken using a Hitachi HT770 at a 100 kV accelerating voltage. Raman spectra were recorded on a WITec Raman microscope alpha300 R. The conductivity of the conductive leather was measured using a standard four‐point probe method at room temperature. The current differences and the *I*–*V* characteristics for the pressure sensor were recorded by the Keithley 2450 and 4200. The pressure measurements were performed on a mechanized *z*‐axis stage (ESM303 Mark 10), and a force gauge (M5‐10 Mark‐10) was used to apply loads to a 4 cm^2^ pressure sensitive pad. The e‐skin was tested on a volunteer (T. Zhang, co‐author) with his consent.

## Conflict of Interest

The authors declare no conflict of interest.

## Supporting information

Supplementary
