# Supplementary material for: Repurposed Leather with Sensing Capabilities for Multifunctional Electronic Skin
Source: Adv Sci (Weinh). 2018 Dec 1;6(3):1801283. doi: 10.1002/advs.201801283 (PMC6364595; doi:10.1002/advs.201801283)
Supplement: Supplementary file 1 — Supplementary [file ADVS-6-1801283-s001.pdf]

Copyright WILEY-VCH Verlag GmbH & Co. KGaA, 69469 Weinheim, Germany, 2018.

ADVANCED SCIENCE
Open Access

Supporting Information

for *Adv. Sci.,* DOI: 10.1002/advs.201801283

Repurposed Leather with Sensing Capabilities for Multifunctional Electronic Skin

Binghua Zou, Yuanyuan Chen, Yihan Liu, Ruijie Xie, Qinjie Du, Tao Zhang, Yu Shen, Bing Zheng, Sheng Li, Jiansheng Wu, Weina Zhang, Wei Huang, Xin Huang,* and Fengwei Huo**

Supporting Information

Repurposed Leather with Sensing Capabilities for Multifunctional Electronic Skin

Binghua Zou[1], Yuanyuan Chen[1], Yihan Liu[1], Ruijie Xie[1], Qinjie Du[1], Tao Zhang[1], Yu Shen[1], Bing Zheng[1], Sheng Li[1], Jiansheng Wu[1], Weina Zhang[1], Wei Huang[1,3*], Xin Huang[2*], and Fengwei Huo[1*]

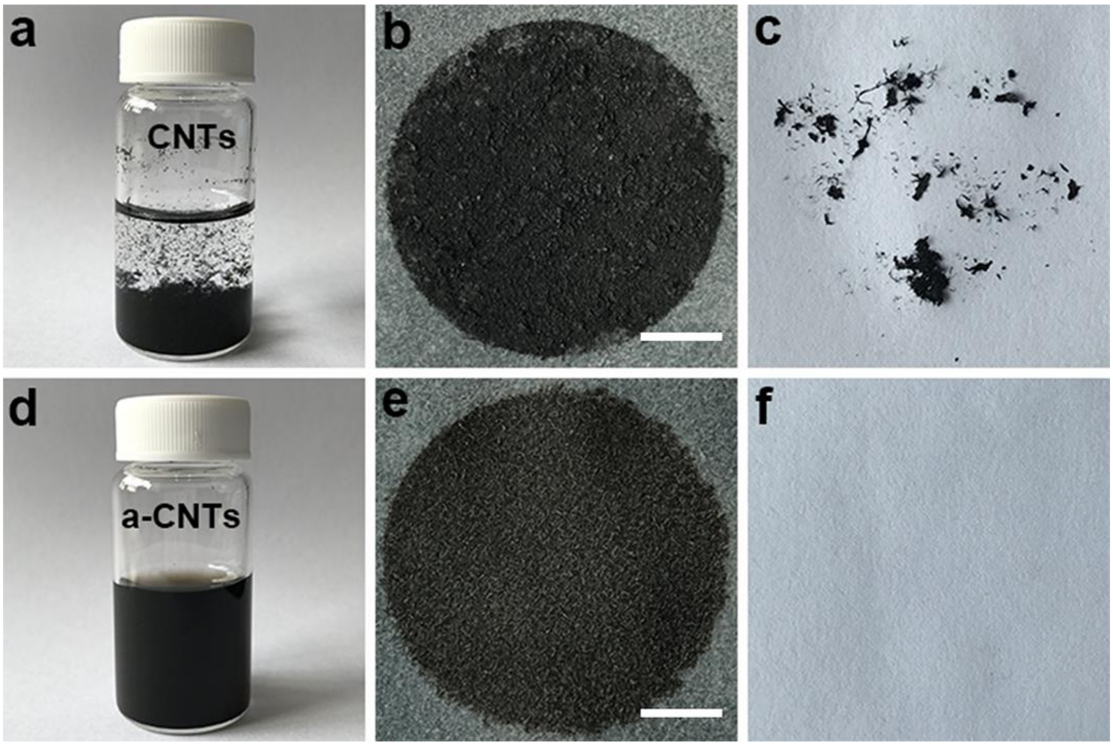

Figure S1. Control experiments of leather filtrated by CNTs and a-CNTs. a) Photographs of CNTs aqueous solution. b) Photographs of leather after filtrating by CNTs aqueous solution. c) Photographs of paper after touching with the CNTs filtrated leather. d) Photographs of a-CNTs aqueous solution. e) Photographs of leather after filtrating by a-CNTs aqueous solution. f) Photographs of paper after touching with the a-CNTs/Leather. Scale bars in b, e), 1 cm.

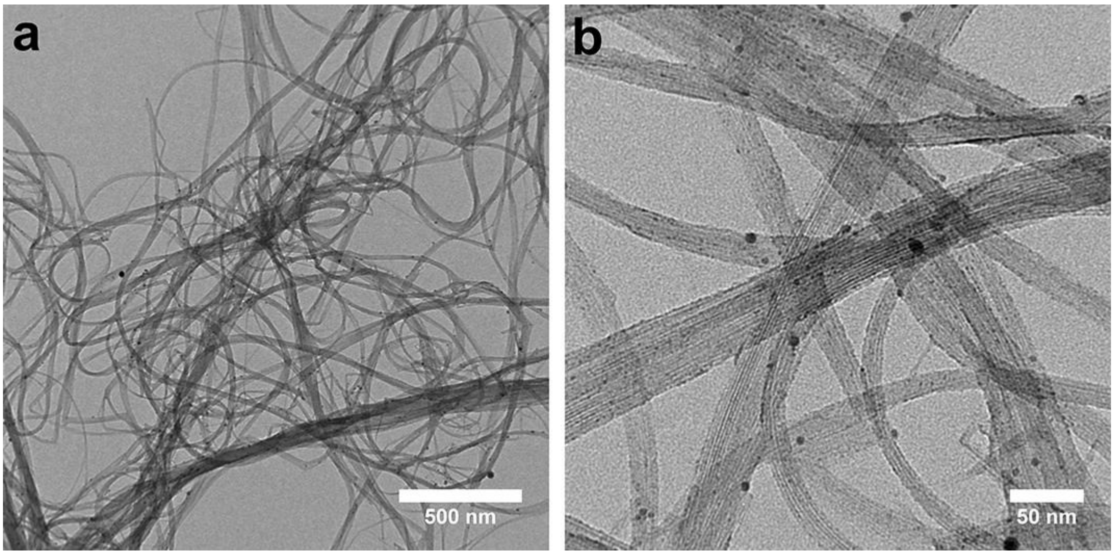

Figure S2. TEM images of a-CNTs. Images showed a-CNTs with micrometers in length and about 30 nm in width.

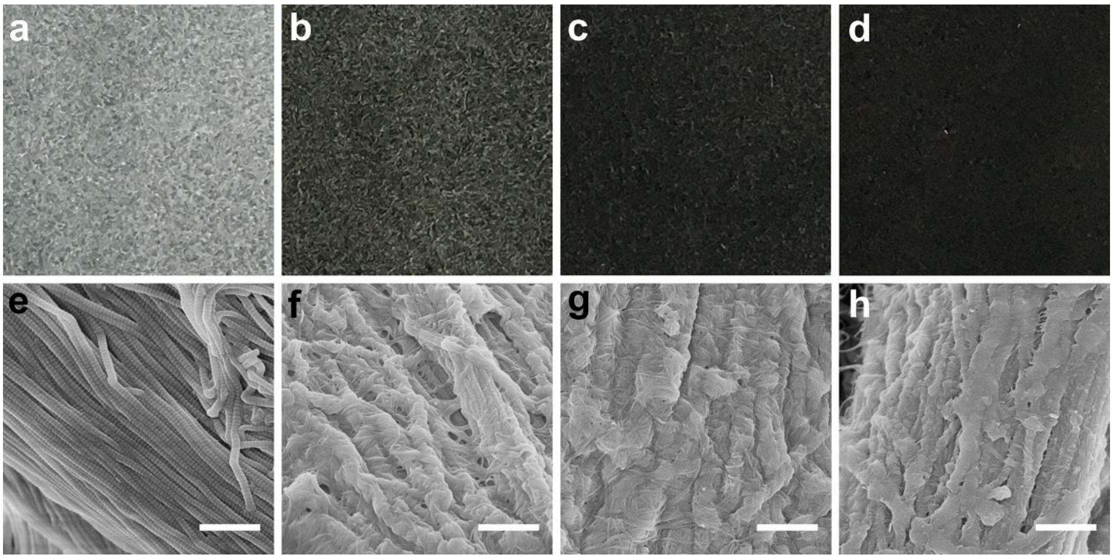

Figure S3. Conductive leathers with different usage amount of a-CNTs. a, e) Photograph and SEM image of leather. b-d) Photographs and f- h) SEM images of conductive leather with increasing the usage amount of a-CNTs. All scale bars are 1 μm.

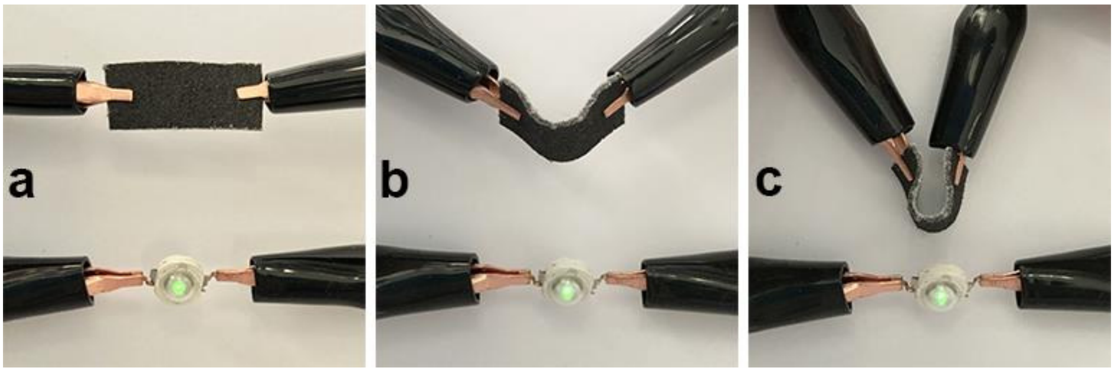

Figure S4. Conductive leather maintain good conductivity when be bent into different angles.

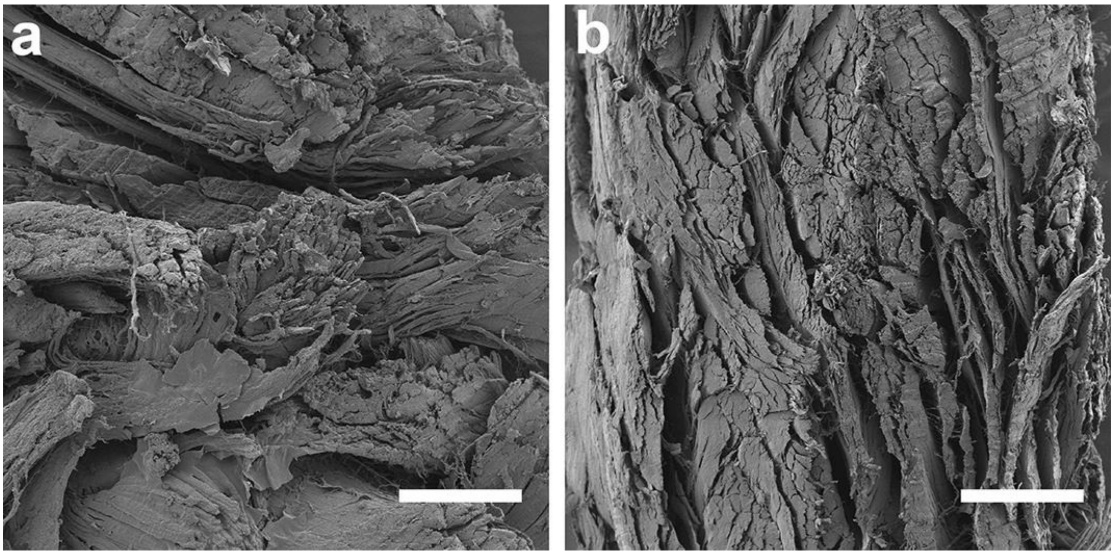

Figure S5. Hierarchical and porous structure of leather. a) SEM image of the surface of leather (scale bar, 100 μm). b) SEM image of the cross section of leather (scale bar, 200 μm).

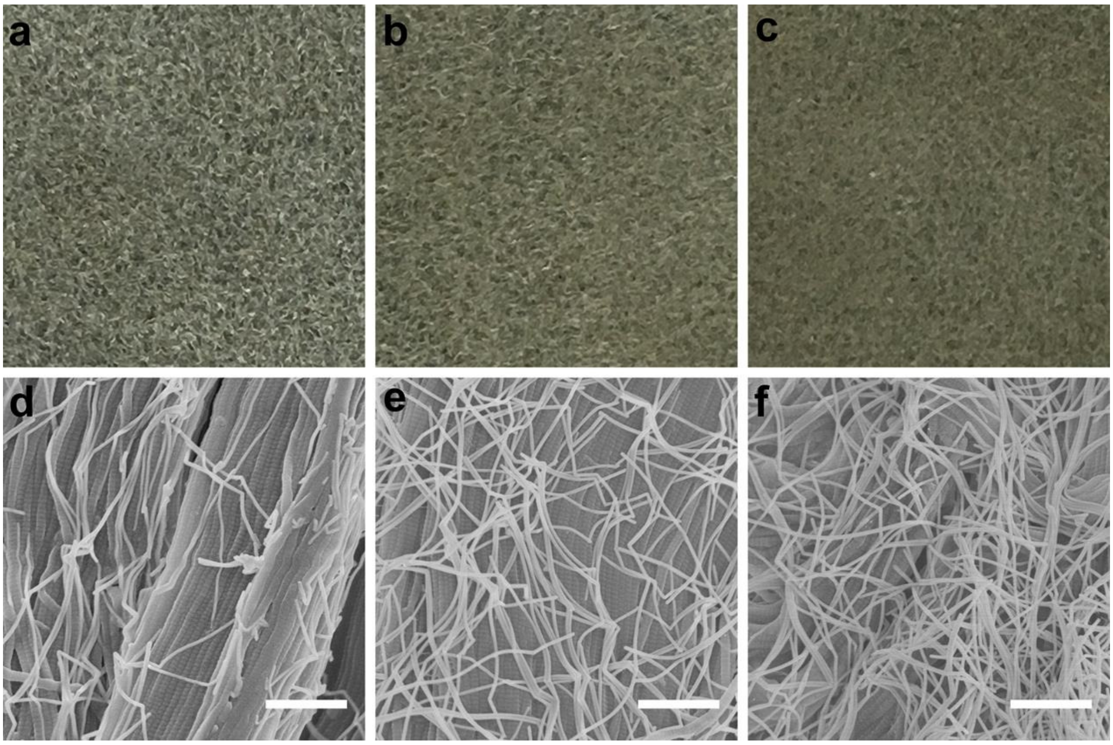

Figure S6. Conductive leathers with different usage amount of Ag NWs. a-c) Photograph and d-f) SEM images of conductive leather with increasing the usage amount of Ag NWs. All scale bars are 1 μm.

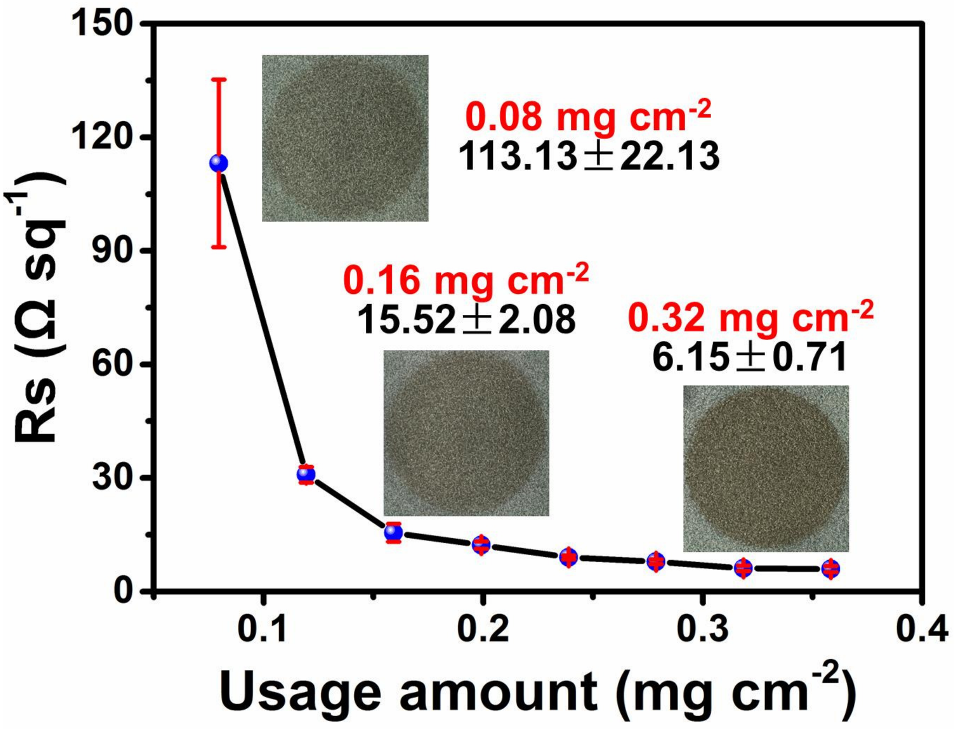

Figure S7. Leather with tunable conductivity filtrated by Ag NWs. Inset photographs are leather after filtrating with different usage amounts of Ag NWs.

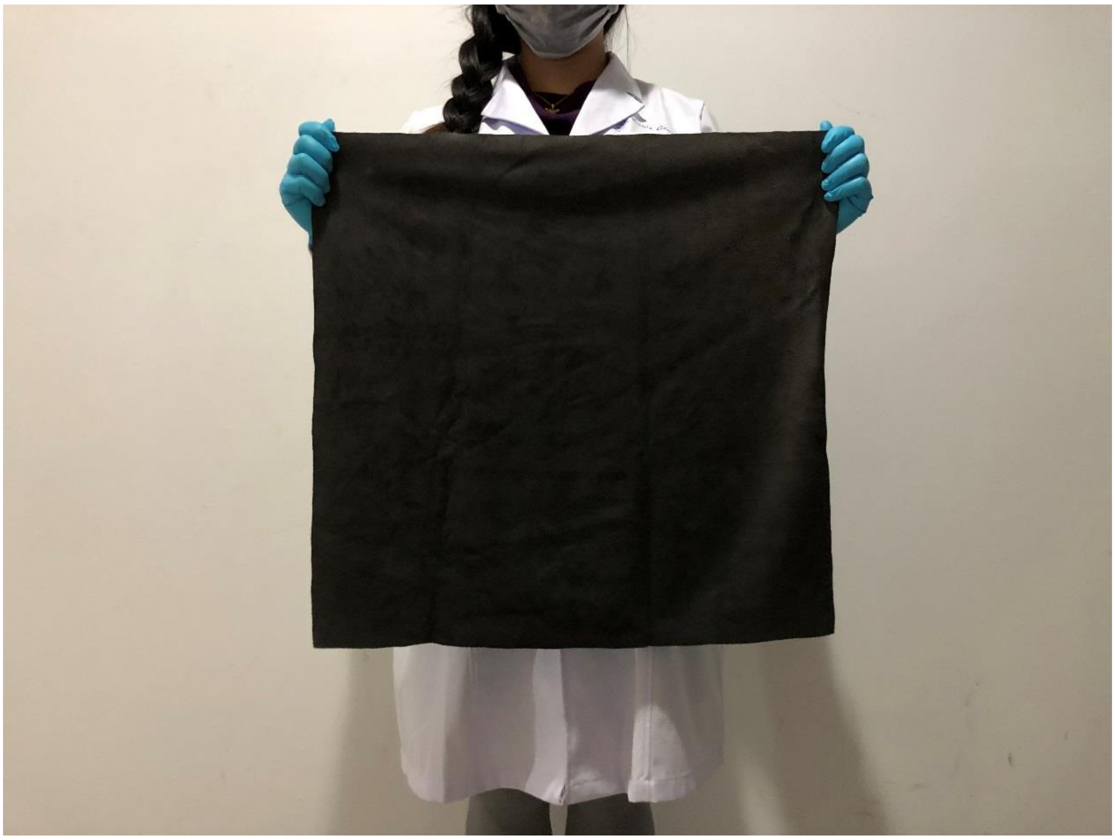

Figure S8. Photograph of 50 cm × 50 cm leather fabricated by tanning procedure in leather industry.

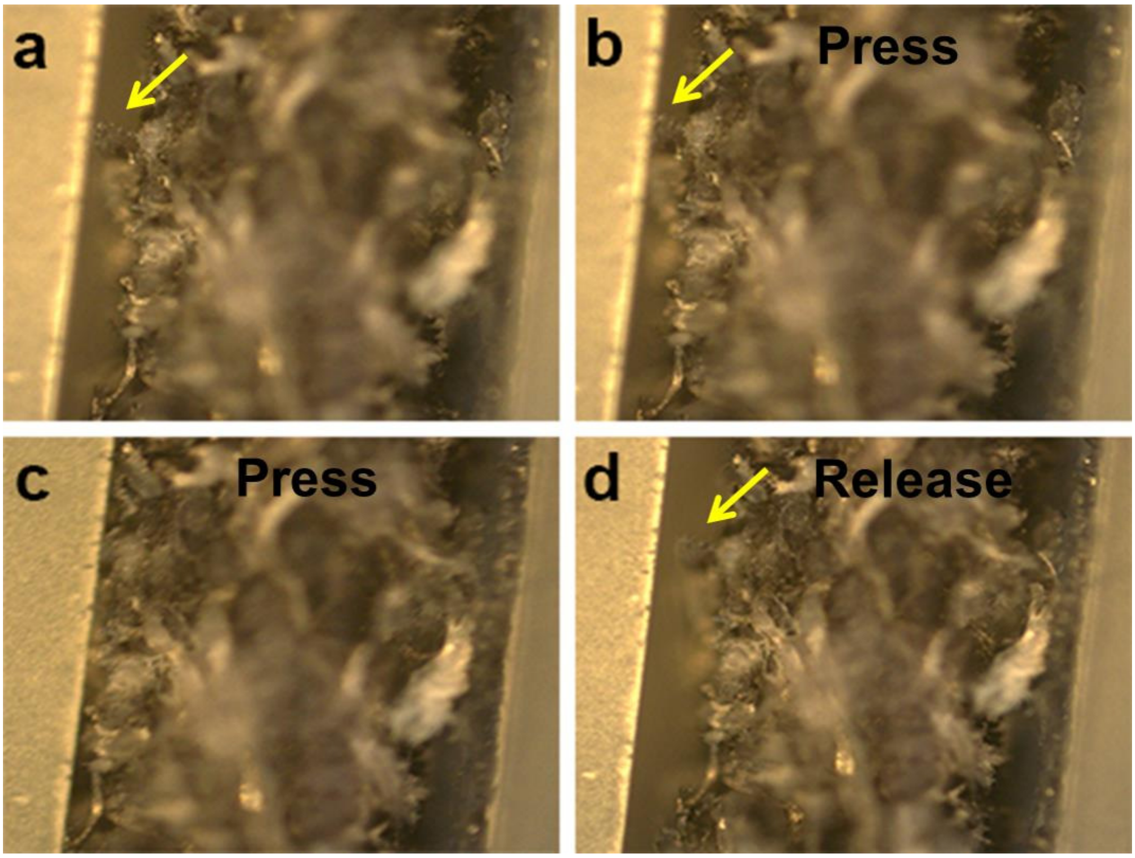

Figure S9. Photographs of a-CNTs/Leather under different forces.

Table S1. Leather based e-skin in comparison with previously reported e-skin based on other materials.

Strategies	PDMS microstructure based e-skin [29-31, 33]	Paper-based pressure sensor [22]	Nanocomposite based e-skin [28]	Leather based e-skin This work
Fabrication Procedure	Photolithography	Impregnation	Synthesis	Filtration (Natural structure)
Sensitivity (< 1 kPa)	8.4 kPa^{-1}	1.14 kPa^{-1}	0.0067 kPa^{-1}	8.03 kPa^{-1}
Substrate	PDMS	Tissue paper-PDMS	Polyimine	Leather (Comfort to wear)
Other Functions			Multifunctional	32.42 kPa^{-1} (< 200 Pa); Multifunctional; Scalable

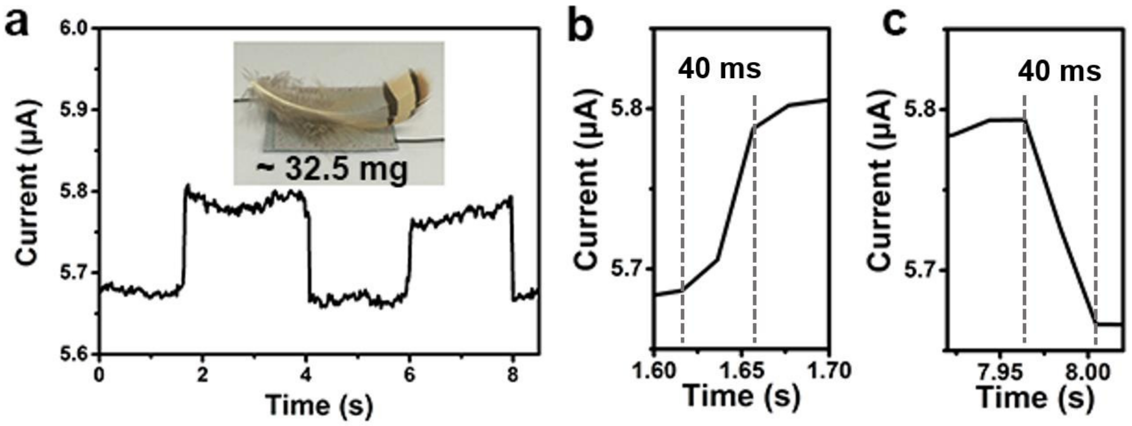

Figure S10. Pressure sensor immediately responded to a light feather. a) Optical image and current curve of our sensor pressed by a light feather. b, c) Corresponding responds time and delay time.

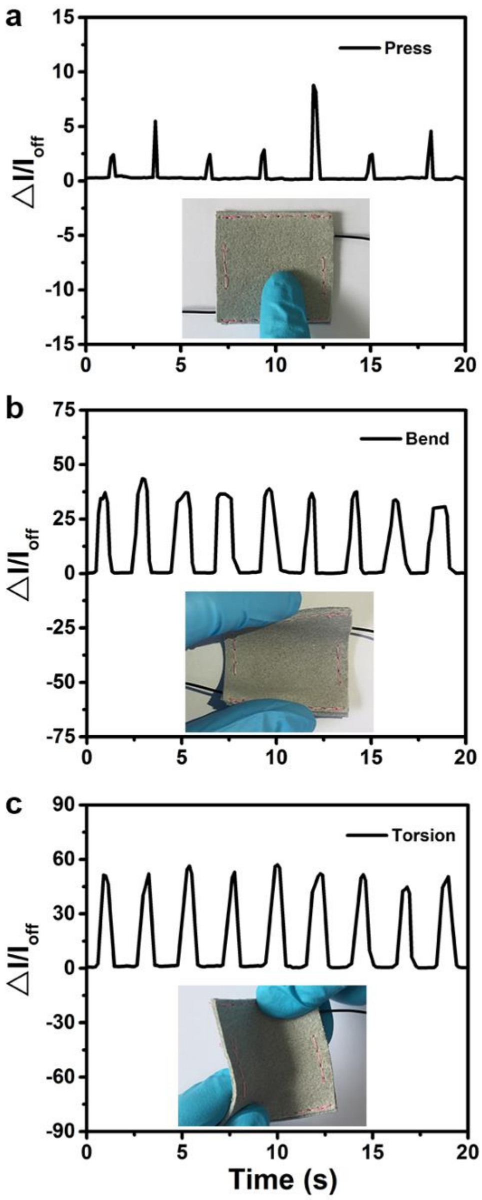

Figure S11. Detection of other types of mechanical forces by conductive leather based pressure sensor. a-c) Plots showing the current responses to dynamic loading and unloading cycles: a) pressing, b) bending and c) torsion.

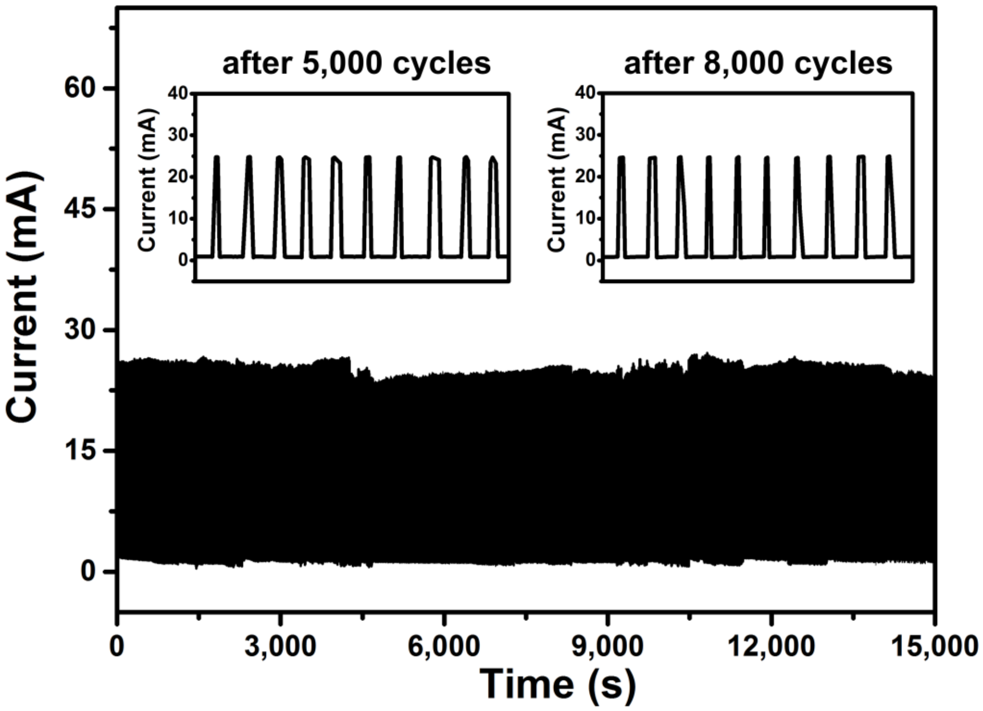

Figure S12. The cycling durability test of pressure sensor for more than 8,000 loading/unloading cycles, with an applied pressure of 2.5 kPa.

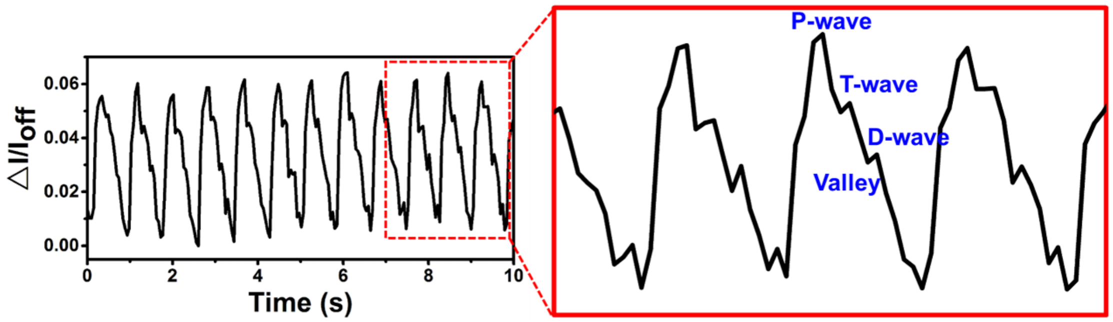

Figure S13. A typical characteristics of wrist pulses. It clearly contains percussion wave (P-wave), tidal wave (T-wave), Valley, and diastolic wave (D-wave).

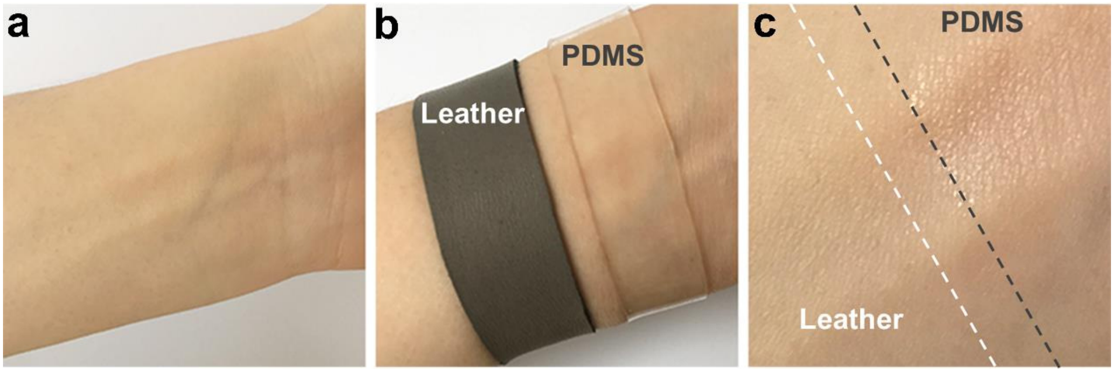

Figure S14. Comfortable control experiments between leather and PDMS. a) Wrist before wearing leather and PDMS. b) Wrist wearing leather and PDMS. c) Wrist after wearing leather and PDMS for 4 h.

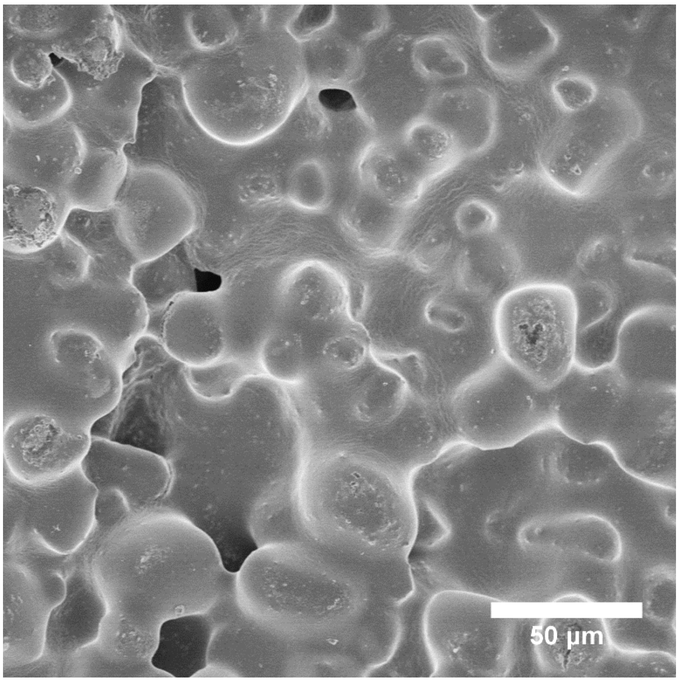

Figure S15. Commercial available ZnS:Cu electroluminescence after coating on the surface of conductive leather.

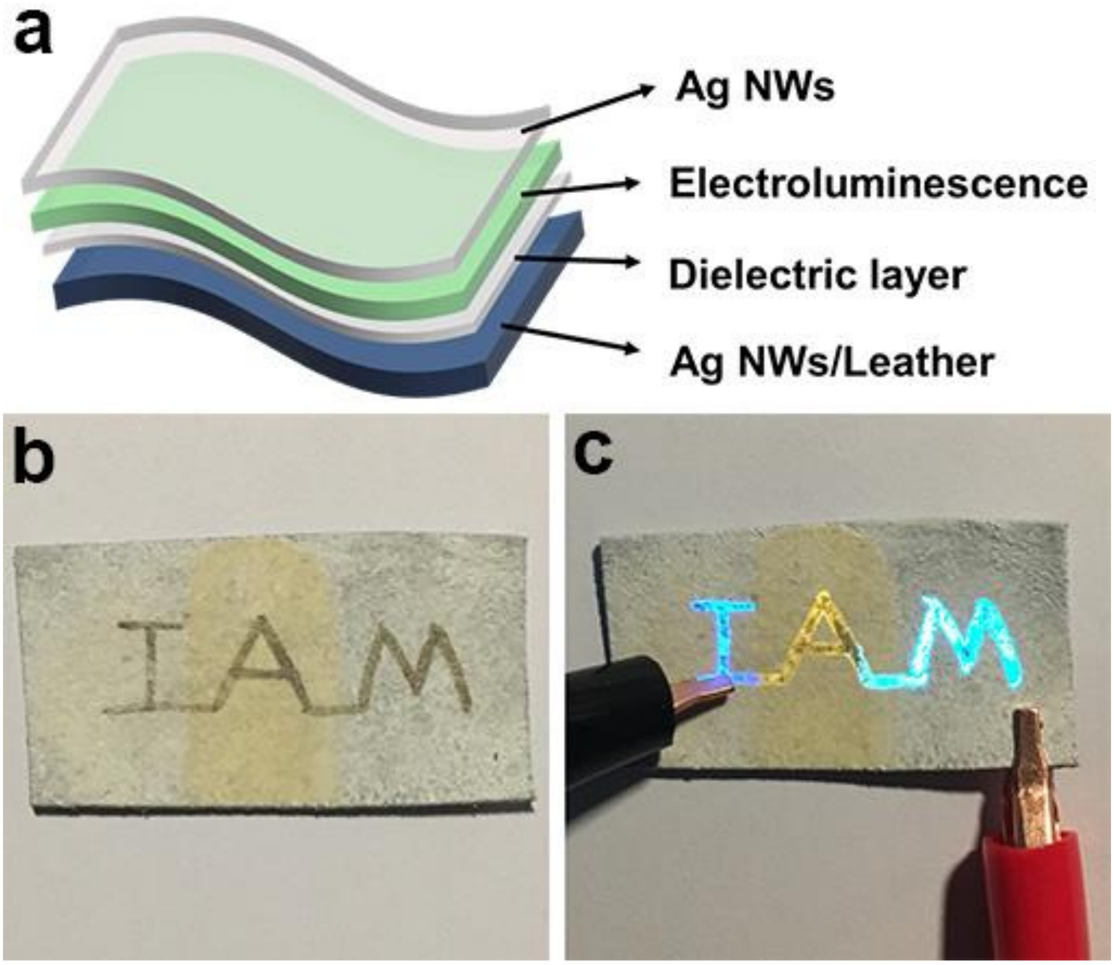

Figure S16. Display based on the Ag NWs filtrated leather. a) The structure of Ag NWs/Leather based display. b, c) The device exhibits a bright color as the power on.

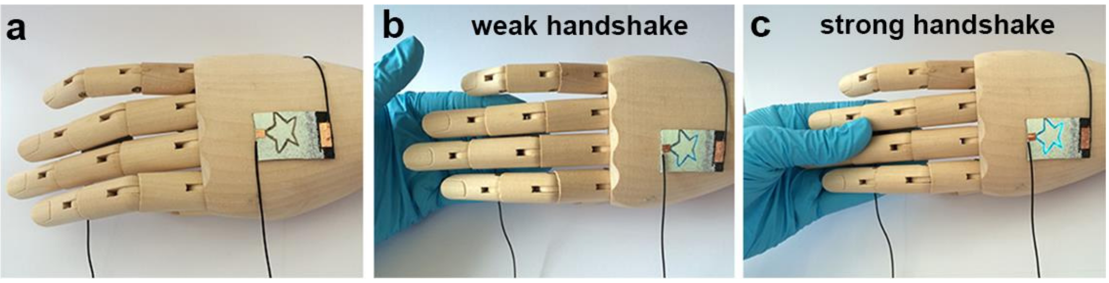

Figure S17. Integration of display and pressure sensor. The brightness of “star” changed under different pressure loading.

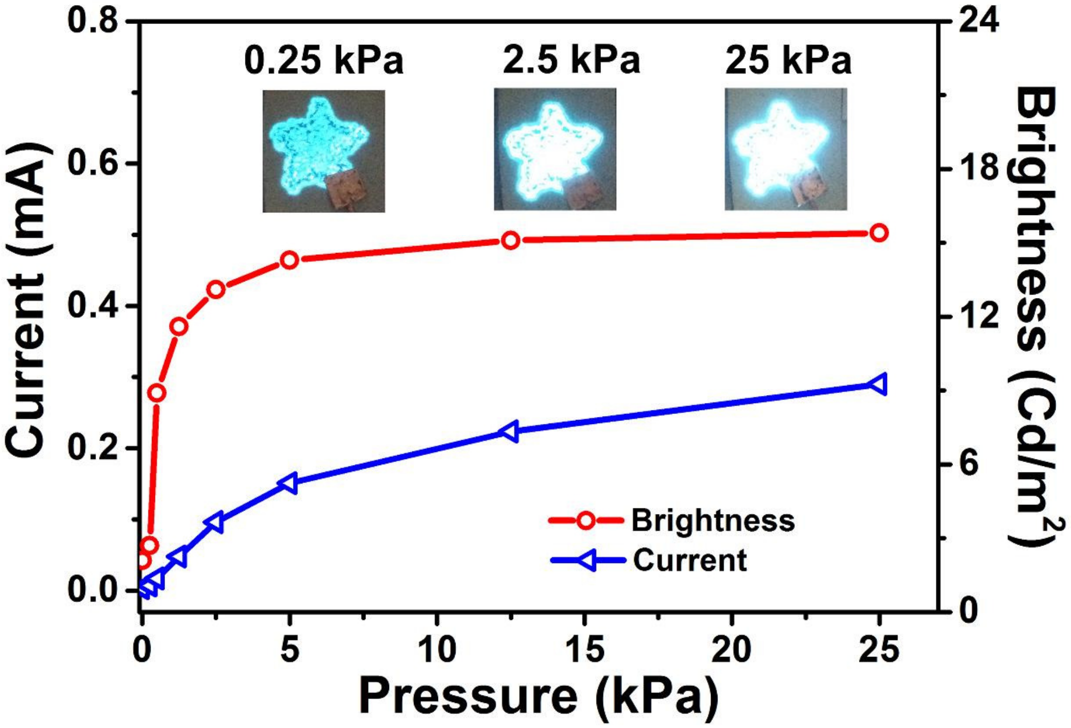

Figure S18. Pressure response of e-skin with display. The current (blue trace) and brightness (red trace) of the display as a function of applied pressure. Inset: photographs of a display under various applied pressures.

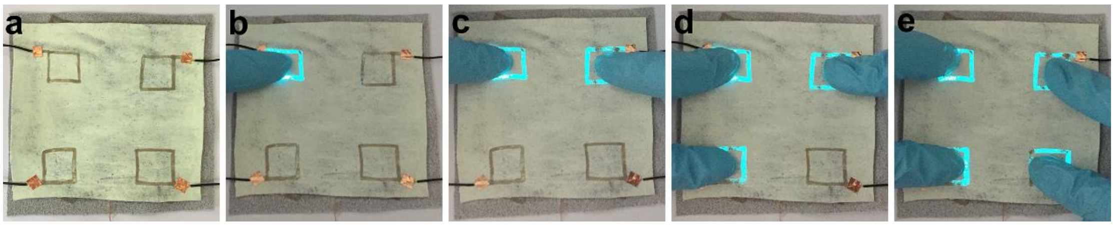

Figure S19. Interactive e-skin. It can spatially map and respond to the applied pressure in form of light.

References

22. S. Gong, W. Schwalb, Y. Wang, Y. Chen, Y. Tang, J. Si, B. Shirinzadeh, W. Cheng, Nat. Commun. **2014**, 5, 3132.

28. Z. Zou, C. Zhu, Y. Li, X. Lei, W. Zhang, J. Xiao, Sci. Adv. **2018**, 4, eaaq0508.

29. S. C. B. Mannsfeld, B. C. K. Tee, R. M. Stoltenberg, C. V. H. H. Chen, S. Barman, B. V. O. Muir, A. N. Sokolov, C. Reese, Z. Bao, Nat. Mater. **2010**, 9, 859.

30. D. J. Lipomi, M. Vosgueritchian, B. C. Tee, S. L. Hellstrom, J. A. Lee, C. H. Fox, Z. Bao, Nat. Nanotechnol. **2011**, 6, 788.

31. G. Schwartz, B. C. K. Tee, J. Mei, A. L. Appleton, D. H. Kim, H. Wang, Z. Bao, Nat. Commun. **2013**, 4, 1859.

33. S. Wang, J. Xu, W. Wang, G. N. Wang, R. Rastak, F. Molina-Lopez, J. W. Chung, S. Niu, V. R. Feig, J. Lopez, T. Lei, S. K. Kwon, Y. Kim, A. M. Foudeh, A. Ehrlich, A. Gasperini, Y. Yun, B. Murmann, J. B. Tok, Z. Bao, Nature, **2018**, 555, 83.
